# Development of a quadruple PCR-based gene microarray for detection of vaccine and wild-type classical swine fever virus, African swine fever virus and atypical porcine pestivirus

**DOI:** 10.1186/s12985-022-01933-9

**Published:** 2022-11-29

**Authors:** Ying-ju Xia, Lu Xu, Jun-jie Zhao, Yuan-xi Li, Rui-zhi Wu, Xiang-peng Song, Qi-zu Zhao, Ye-bing Liu, Qin Wang, Qian-yi Zhang

**Affiliations:** China Institute of Veterinary Drug Control, Beijing, 100081 People’s Republic of China

**Keywords:** Classical swine fever, African swine fever, Atypical pestivirus, Polymerase chain reaction, Differential diagnosis, Gene chip

## Abstract

**Background:**

Classical swine fever (CSF), African swine fever (ASF), and atypical porcine pestivirus (APPV) are acute, virulent, and contagious viral diseases currently hampering the pig industry in China, which result in mummification or stillbirths in piglets and mortality in pigs. Diagnostic assays for the differentiation of infection and vaccination of CSFV, in addition to the detection of ASFV and APPV, are urgently required for better prevention, control, and elimination of these viral diseases in China.

**Methods:**

A quadruple PCR-based gene microarray assay was developed in this study to simultaneously detect wild-type and vaccine CSFV strains, ASFV and APPV according to their conserved regions. Forty-two laboratory-confirmed samples, including positive samples of 10 other swine viral diseases, were tested using this assay to confirm its high specificity.

**Results:**

This assay's limit of detections (LODs) for the wild-type and vaccine CSFV were 6.98 and 6.92 copies/µL. LODs for ASFV and APPV were 2.56 × 10 and 1.80 × 10 copies/µL, respectively. When compared with standard RT-PCR or qPCR for CSFV (GB/T 26875–2018), ASFV (MARR issue No.172), or APPV (CN108611442A) using 219 clinical samples, the coincidence was 100%. The results showed that this assay with high sensitivity could specifically distinguish ASFV, APPV, and CSFV, including CSFV infection and immunization.

**Conclusion:**

This assay provides a practical, simple, economic, and reliable test for the rapid detection and accurate diagnosis of the three viruses and may have good prospects for application in an epidemiological investigation, prevention, and control and elimination of these three diseases.

## Introduction

Classical swine fever (CSF) is an acute, febrile, highly contagious, and lethal infectious disease caused by classical swine fever virus (CSFV) belonging to the Pestivirus genus, *Flaviviridae* family [1]. It is an World Organization of Animal Health (WOAH) notified animal disease and defined as China's highly pathogenic microorganism [2]. It naturally infects domestic pigs and wild boars only despite age, gender, species, and seasons [3]. African swine fever (ASF) is an acute, hemorrhagic, lethal disease caused by African swine fever virus (ASFV), which is also an WOAH-notified disease [4]. It is characterized as short onset, 100% lethal in most acute and acute cases [5]. Atypical porcine pestivirus (APPV), also known as congenital tremor or “dancing piglet”, resulted in paroxysmal contracture in the head, legs, and other parts of body muscles in piglets, which consequently caused piglets to lose the ability to stand and suck milk, even to die [6, 7]. It was first reported in 2017 in Guangdong Province in China and subsequently reported in piglets in other places, which indicated its epidemic in China [8]. Currently, the above three diseases are epidemic in China [9–11]; therefore, a fast and differential diagnosis of these three diseases in clinical is important.

In addition, prevention and control of CSF in China mainly rely on vaccination with the C strain, contributing to CSF control globally [12]. However, chronic and atypical infections still occur, challenging CSF prevention and control [13]. Owing to the similarity of classical live attenuated vaccines and wild-type strains of CSFV, differentiation diagnosis between infected and vaccinated animals for CSFV is still unavailable in China. Regarding ASFV and APPV, no effective vaccines or treatments are available. Therefore, an assay simultaneously detecting CSFV, ASFV, and APPV in this scenario will almost certainly fascinate the diagnosis, prevention, and control of those diseases.

Conventional laboratory diagnoses for CSFV and ASFV referred to WOAH manual are virus isolation, RT-PCR/PCR, ELISA, and fluorescent antibody test, among which the real-time PCR method is most widely used as for its high sensitivity, rapid completion, and cost-effectiveness. Recently, gene microarray has been widely used in medical science and has achieved outstanding results in the research of gene expression, pathogenesis, clinical diagnosis, drug development, and biological detection [14, 15]. Previous studies have developed microarray assays for the simultaneous detection of avian respiratory viral diseases, including avian influenza, Newcastle disease, and infectious bronchitis virus [16]. A similar system was also established for the detection of seven cattle pathogens. So far, the microarray developed for detecting CSFV, ASFV and APPV have not been reported yet [17]. In this study, a gene microarray assay was developed to distinguish these three pathogens, including wild-type and vaccine CSFV strains, respectively. Three pairs of primers and corresponding probes were designed based on the conserved region of CSFV, ASFV and APPV to establish a reliable and rapid gene microarray assay for the differential diagnosis of ASFV, APPV, and CSFV, which will be useful for clinical diagnosis as well as epidemiological investigation of these diseases in large-scale pig farms.

## Materials and methods

### Viruses and clinical samples

Wild-type CSFV and vaccine strains (C strain and Thiversal strain) used in this study were isolated and storied by National Reference Laboratory for CSF at the Institute of China Veterinary Drug Control (IVDC). Japanese encephalitis virus (JEV), bovine viral diarrhea virus (BVDV), porcine reproductive and respiratory syndrome virus (PRRSV), porcine epidemic diarrhea virus (PEDV), transmissible gastroenteritis virus (TGEV), porcine circovirus 1 (PCV1), porcine circovirus 2 (PCV2), porcine parvovirus (PPV) and pseudorabies virus (PRV) were provided by China Animal Disease Control Center (CADC). ASFV-positive samples were disinfected and provided by the Harbin Veterinary Research Institute (HVRI) of the Chinese Academy of Agricultural Sciences (CAAS). APPV clinical samples were kindly provided by Dr. Xu Zhiwen from Sichuan Agricultural University (SAU). Detailed information on these samples is shown in Table 1.

**Table 1:** Viruses information

Virus	Sample name	Sample number	Virus	Sample name	Sample number
CSFV	Shimen	1	CSFV	HBJZ1	22
	BJYQ1	2		LNCY1	23
	SX4	3		SCMY1	24
	HeBHD2	4		HBXY4	25
	HeBHH1	5		HBXY5	26
	HeBBD1	6		ZYBJ1	27
	TJNH1	7		HeBBD4	28
	HeBJZ1	8		Thiveosal strain	29
	HeBQHD1	9		Chinese strain	30
	HeNZZ1	10	ASFV	HuBES4	31
	HBES2	11	APPV	SCMY1	32
	ZJHZ1	12		FMDV	33
	HENZMD1	13		BVDV	34
	HeNXC3	14		PCV-1	35
	JSXZ1	15		PCV-2	36
	GXFL1	16	Other	PRV	37
	HeBCB2	17		PPV	38
	HENZMD2	18		PRRSV	39
	HeNXC1	19		TGDV	40
	HBHG1	20		PEDV	41
	HBHM1	21		JEV	42

### Primers and probes

Based on the gene sequences of CSFV wild type (CSFV-W) and vaccine strains (CSFV-V) published in GenBank, as well as the reference strains of ASFV and APPV, five pairs of specific primers with a biotin tag and the corresponding gene microarray probes were designed by our laboratory and published in previous studies [18, 19]. The 5′UTR is the most conserved region for CSFV, which was used for primer design as a universal detection target for CSFV. The NS5B gene in the vaccine strains has a one-base difference compared to CSFV-W, and the primer and probe were designed only to amplify CSFV-V based on an amplification refractory mutation system PCR principle (ARMS-PCR). The conserved B646L (encoding p72) gene and the conserved 5′UTR of porcine APPV were selected for the primer and probe design of ASFV and APPV, respectively. Beta-globin gene (GenBank: AH001475.2) was selected for the primers and probe design for PCR internal control. Detailed information on primers and probes used in this study can be found in Tables 2 and 3.

**Table 2:** Primers of CSFV, ASFV, APPV and beta-globin used in this study

Name of Primers^a^	Primer sequence (5′ → 3′)	Target gene	Product size (bp)
CSFV-W-F	GGAGGGACTAGCCRTAGTG	5′UTR	77
CSFV-W-R	ACGTCGAACTACTGACGACTG-biotin		
CSFV-V-F	CCTTCGGGGAGAAAGTAACGAT	NS5B	97
CSFV-V-R	CCTACCACAGTCACGGCT-biotin		
ASFV-F	TATATTGGCCCAAGACTTGCT	B646L	119
ASFV-R	GCACCAAATGTGTTTCTTCGAT-biotin		
APPV-F	CAGACGTCACCGAGTAGTACACC -biotin	5′UTR	134
APPV-R	CCCAGGTCCACCACCGAT		
IC-F	AAGTCTGCCGTTACTGCC-biotin	Beta-globin	83
IC-R	TAACCTTGATACCAACCTGC		

^a^*CSFV-W-P*: probe for detection of wild type CSFV; *CSFV-V-P*: probe for detection of CSFV vaccine strains

**Table 3:** Probes used in this study

Name of Probes^a^	Probe sequence (5′ → 3′)	Length of the probe (bp)
CSFV-W-P	CCCTGGGTGGTCTAAGTCCTGAGTACAG	29
CSFV-V-P	ATGCAGGAGGAGATAACCTTGCAGCC	26
ASFV-P	AACCCGATCCCGAACCCACT	20
APPV-P	ATGCCCACGTCCACCCAAGCC	21
IC-P	CCACCAACTTCATCCACGTTCACC	24

Biotin has been attached to the primers. Activated streptavidin (SA) and horseradish peroxidase (HRP) are covalently conjugated to the membrane of the microarray. When primers have specifically amplified the expected PCR product, the product can hybridize with probes on the microarray, and biotin can interact with SA-HRP. Subsequently incubated with TMB (3,3′,5,5′-Tetramethylbenzine) which can produce a deep blue color during the enzymatic degradation of hydrogen peroxide by HRP and determine the results.

^a^CSFV-W-P: probe for detection of wild type CSFV; CSFV-V-P: probe for detection of CSFV vaccine strains; ASFV-P: probe for detection of  ASFV; APPV-P: probe for detection of  APPV;IC-P: probe for detection of beta-globin gene.Sequences used for designing primers and probes were obtained from GenBank. GenBank accession numbers were as follows: AF531433.1 (CSFV/HCLV), AY775178.2 (CSFV/Shimen/HVRI), KR233071.1 (CSFV/HuN23/2013), AY259122.1 (CSFV /Riems), GU324242.1 (CSFV/Uelzen); MK333180.1 (ASFV/Pig/HLJ/2018), NC_001659.2 (ASFV/ BA71V), MW183242.1 (APPV/USA/2017), MN080493 (APPV/China/2017), KY475593.1 (APPV/China/2016), AH001475.2(beta-globin gene).

### Development of the quadruple PCR

ASFV viral DNA, CSFV, and APPV viral RNAs were extracted according to manufactory’s instruction (TaKaRa MiniBEST Viral RNA/DNA Extraction Kit Ver.5.0, Cat:9766). A quadruple one-step RT-PCR was developed as follows: PrimeScript (TaKaRa Bio, China) one step Enzyme Mix 1 μL, 2 × Super Multiplex PCR Mix 12.5 μL, five pair of primers 1 μL each, RNA/DNA template 4 μL each, IC plasmid 1 μL. Sterilized double distilled water (ddH_2_O) up to 25 μL. The annealing temperature (56 °C, 58 °C, 60 °C, 62 °C, 64 °C), the concentration of primers (final concentration 3.2 pmol/μL, 2.4 pmol/μL, 1.6 pmol/μL, 0.8 pmol/μL) and extension period were optimized respectively to get an efficient and time-saving PCR assay. This assay also set up internal and external control. The final optimized PCR composition and condition were as follows: a master mix of 25 μL reaction is composed of 1 μL of Primscript RT Master Mix, 12.5 μL of 2 × Super Multiplex PCR Mix, 1 μL of each primer (final concentration was 3.2 pmol/μL)1 μL of IC plasmid, 1 μL of template DNA, 3.5 μL of ddH20. The parameters for quadruple RT-PCR start with reverse transcription at 37 °C for 15 min, followed by inactivation of reverse transcriptase at 85 °C for 10 s; then with a denaturation step at 95 °C for 2 min, followed by 35 cycles of denaturation at 98 °C for 15 s, annealing/extension at 60 °C for the 20 s. PCR products then store at 4 °C for later use.

### Preparation for microarray

**Fig. 1:**
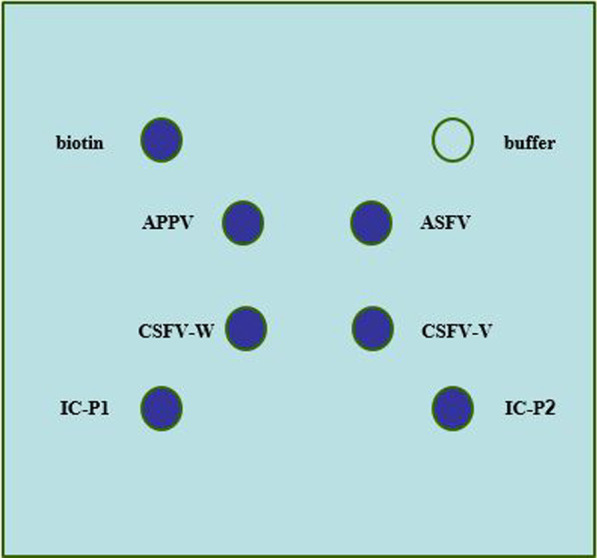
The diagram of the chip spot pattern

The 2% EDC (1–3-dimethylaminopropyl)-3-ethylcarbodiimide hydrochloride, Sinopec Qilu, China) and the 1.21% NHS(w/v,CN-hydroxysuccinimide, Shanghai Covalent Chemical Tech, China) were fully dissolved in ultrapure water for preparation of the activation solution. 76 mm × 65 mm modified silica membranes (Hubei Huifu Nanomaterials, China) were placed in an activation bath with the front side up and 15 ml per membrane activation solution was poured evenly into the bath to immerse the membrane surface for 30 min. Subsequently, the membrane was washed three times with ultrapure water and blown dry with nitrogen to ensure the membrane surface was dry and clean. The activated membranes were loaded onto the membrane rack to assemble a 48-well plate which was then loaded into the corresponding positions on the spotter. The probes were diluted to a final concentration of 6 µM, and 4 µL was added to each well. The parameter of the spotter was set to 100 drops, and the probes were dispensed onto the 48-well assembly of modified silica membranes according to the pre-arranged dispensing sequence to assemble the gene chips. The diagram of the chip spot pattern is shown in Fig. 1.

Specific probes were dispensed onto each well, as demonstrated in the diagram. The outside four spots were negative and positive controls: Biotin is the control for monitoring the efficient hybridization on the microarray. IC-P is a control for monitoring the efficient PCR progress and indicates the direction for results interpretation. APPV, ASFV, CSFV-W, and CSFV-V were dispensed inside.

### Design and composition of gene chip

Preparation of hybridization buffer A, elution buffer B and rinse buffer C as follows. Hybridization buffer A: mixed up 10 mL of 20 × SSC and 1 mL of 10% SDS into purified water up to 100 mL. Elution solution B: took 3 mL of 20 × SSC and 1.2 mL of 10% SDS, purified water to 120 mL, and mixed thoroughly. Rinse solution C: took 10 mL of 1 mol sodium citrate into purified water to 100 mL and mixed thoroughly. All three buffers were stored at room temperature for up to 6 months.

We preheated the chip and buffer B at 47 °C. Mixed 10 μL of PCR product with 110 μL of buffer A and added to the chip at 120 μL/well at 47 °C 200 r/min for 5 min. The chip was washed three times with pre-warmed buffer B (100 μL/well). SA-HRP (Streptavidin Conjugated with Horseradish Peroxidase, Sigma, USA) was diluted at the ratio of 1:2000 with buffer A, then added to the wells with 100 μL/well, followed by 47 °C 200 r/min incubation for 5 min. The chip was washed twice with buffer A (100 μL/well), and then twice with buffer C (100 μL/well) at room temperature. 60 μL/well of TMB (3,3′,5,5′-Tetramethylbenzidine, Sigma) was added and incubated at dark for 1 min, and washed twice with purified water (200 μL/well) and blown dry gently with a compressed air canister for final results interpretation.

### Result interpretation

The test is validated if the biotin spot on the gene chip presented clearly dark blue and at least one of the two IC-P probe spots is colored. If CSFV-W spot is blue, it means the sample is CSFV-W nucleic acid positive. If CSFV-W and CSFV-V spots are both blue, it represents that the sample is CSFV-V nucleic acid positive. If ASFV or APPV spot has no color, it means the sample is ASFV or APPV negative. Otherwise, if blue color shows on the spot, the result is positive.

### Generation of positive control plasmid

Five pairs of specific primers were designed to amplify the 5′UTR of the CSFV-W Shimen strain, the NS5B gene fragment of the CSFV-V C strain, the 5′UTR gene fragment of ASFV/HuBES4 strain, and the 5′ UTR gene fragment of APPV/SCMY1 strain. The PCR products of ASFV/HuBES4 was gel purified and cloned into the pUC57 vector to construct a recombinant plasmid. The remaining three PCR products were gel purified and cloned into the pGEM-T vector, respectively. The recombinant plasmids were sequenced for confirmation, and the sequencing results were analyzed using DNAStar (version 7.1) and NCBI Nucleotide Blast. The correct recombinant plasmids were then used as standard materials and named as CSFV-W-p, CSFV-V-p, ASFV-p, and APPV-p, respectively (Table 4).

**Table 4:** PCR primers for the generation of recombinant plasmids

Name of Primers	Sequence of primer (5′ → 3′)	Product size(bp)	Target gene
CSFV-W-F	GAGGTTAGTTCATTCTCGTATACACGA	310	5′UTR
CSFV-W-R	TATCAGGTCGTACTCCCATCAC		
CSFV-V- F	CCCTTCACAACCTTACCCGACTGATTG	295	NS5B
CSFV-V- R	CAGGCCTGAACCTGAGCTGGTGAAC		
ASFV-F	AGTTATGGGAAACCCGACCC	257	p72
ASFV- R	CCCTGAATCGGAGCATCCT		
APPV-F	CGCGGATCCACAGCCTACTGATGATCAGTCGATG	336	5′UTR
APPV-R	CCGGCACTCTATCAAGCAGTAAGGTC		

### Specificity test

The developed quadruple PCR combined with gene chip assay was used to test the specificity of 42 clinical samples with different swine diseases shown in Table 1, including 28 current circulating CSFV-W strains identified by our laboratory, two CSFV-V strains, ASFV, and APPV samples and 10 other common swine viral disease samples. Sterilized double distilled water was used as a negative control. The specificity of the gene chip was evaluated based on these results.

### Sensitivity test

CSFV-W-p, CSFV-V-p, ASFV-p, and APPV-p were diluted in a tenfold gradient ((10^–8 ^− 10^–1^) for sensitivity test by performing CSFV-W-p, ASFV-p and APPV-p single PCR and gene chip assays, CSFV-(W + V)-p duplex assays and then quadruple assay, respectively. The limits of detection (LOD) of each virus in this assay were calculated using the Dalton copy number formula [copy number = plasmid concentration × 6.02 × 10^23^/(660 × plasmid length)]. The sensitivity of the established quadruple PCR combined with the gene chip assay was evaluated based on the LOD of the single or quadrupled diluted plasmids.

### Tests of clinical samples

To evaluate the accuracy of the gene chip assay for differential diagnosis of field samples, 219 clinical samples (20 spleen, 20 kidney, 20 lymph nodes, and 159 whole blood samples) from a pig farm in Haidian, Beijing (BJHD), Dianjiang, Chongqing (CQDJ), Wanyuan, Sichuan (SCWY) and Baoding, Hebei (HeBBD) and HVRI were tested using this assay, and the results were compared with the results conducted from national standards or published methods including CSFV RT-nPCR assay (GB/T 26875–2018), ASFV real-time qPCR assay (Ministry of Agriculture and Rural Affairs Announcement No. 172), and APPV traditional PCR assay (CN108611442A). Thus, to verify the specificity and sensitivity of this assay again.

## Results

### Construction of standard plasmid as a positive amplification control

The CSFV-W-p, CSFV-V-p, ASFV-p, and APPV-p plasmids were constructed as a positive control for PCR amplification using the specific primers CSFV-W-5′UTR-F/R, CSFV-V-NS5B-F/R, ASFV-p72- F/R, and APPV-5′UTR-F/R. The PCR products were 310 bp for CSFV-W, 295 bp for CSFV-V target gene, 257 bp for ASFV and 336 bp for APPV as expected. Results showed that all the plasmids have the right insertion sequence as their template sequence indicated on NCBI GenBank. The concentration of these plasmids was determined by NanoDrop™ 1000 fluorospectrometer, and the copy number of each plasmid was calculated by Dalton method, which was 3.04 × 10^10^copies/μL, 4.16 × 10^10^copies/μL, 3.81 × 10^10^copies/μL and 4.04 × 10^10^ copies/μL respectively.

### Specificity analysis

**Fig. 2:**
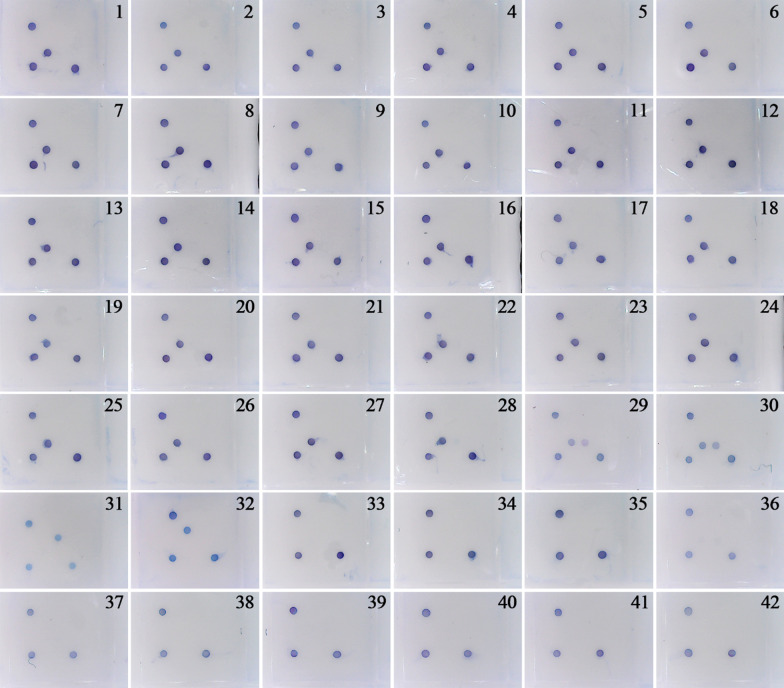
The specificity test results of the gene microarray assay

The results of the gene microarray assay on 42 validated samples showed that the Biotin and IC plasmid control were all in blue, indicating that the assay was valid. 28 CSFV-W strains were had CSFV-W spots in blue but not CSFV-V spots, indicating they were wild-type CSFVs. The two vaccine strains (Chinese strain and Thiveosal strain) showed blue at both CSFV-W and CSFV-V spots indicating they were vaccine strains. The ASFV/HuBES4 and APPV/SCMY1 strains showed blue at ASFV or APPV spots, respectively, demonstrating its success in detecting ASFV and APPV. The other ten swine viral disease samples were all negative for CSFV-W, CSFV-V, ASFV, and APPV, with no color at the four spots, further confirming the high specificity of this assay (Fig. 2).

Forty-two validated samples were tested using this gene microarray assay to determine the assay's specificity.. Each detection well was tested for a sample and recorded as above. The corresponding virus information is as below: 1–28 were wild type CSFV strains showed positive for CSFV wild type; 29–30 were CSFV vaccine strains showed positive both for wild type and vaccine CSFV; 31 was ASFV strain showed positive for ASFV; 32 was APPV strain showed positive for APPV; 33–42 were other swine viruses strain showed negative for CSFV, ASFV, and APPV.

The following is the detailed virus information: 1.CSFV-shimen, 2. CSFV/BJYQ, 13.CSFV/SX4, 4.CSFV/HeBHD2, 5.CSFV/HeBHH1, 6.CSFV/Hebbd1, 7.CSFV/TJNH1, 8.CSFV/HebJZ1, 9.CSFV/JeBQHD1, 10.CSFV/HeHZZ1, 11.CSFC/HBES2, 12.CSFV/ZJHZ1, 13.CSFV/HENZMD1, 14.CSFV/HeNXC3, 15.CSFV/JSXZ1, 16.CSFV/GXFL1, 17.CSFV/HeBCB2, 18.CSFV/HENZMD2, 19.CSFV/HeNXC1, 20.CSFV/HBHG1, 21.CSFV/HBHM1, 22.CSFV/HBJZ1, 23.CSFV/LNCY1, 24.CSFV/SCMY1, 25.CSFV/HBXY4, 26.CSFV/HBXY5, 27.CSFV/ZYBJ1, 28.CSFV/HeBBD4, 29.CSFV/T strain, 30.CSFV/C strain, 31.ASFV/HuBES4, 32.APPV/SCMY1, 33.FMDV, 34.BDVD, 35.PCV1, 36.PCV2, 37.PRV, 38.PPV 39.PRRSV, 40.TGEV, 41.PEDV, 42.JEV.

### Sensitivity analysis

**Fig. 3:**
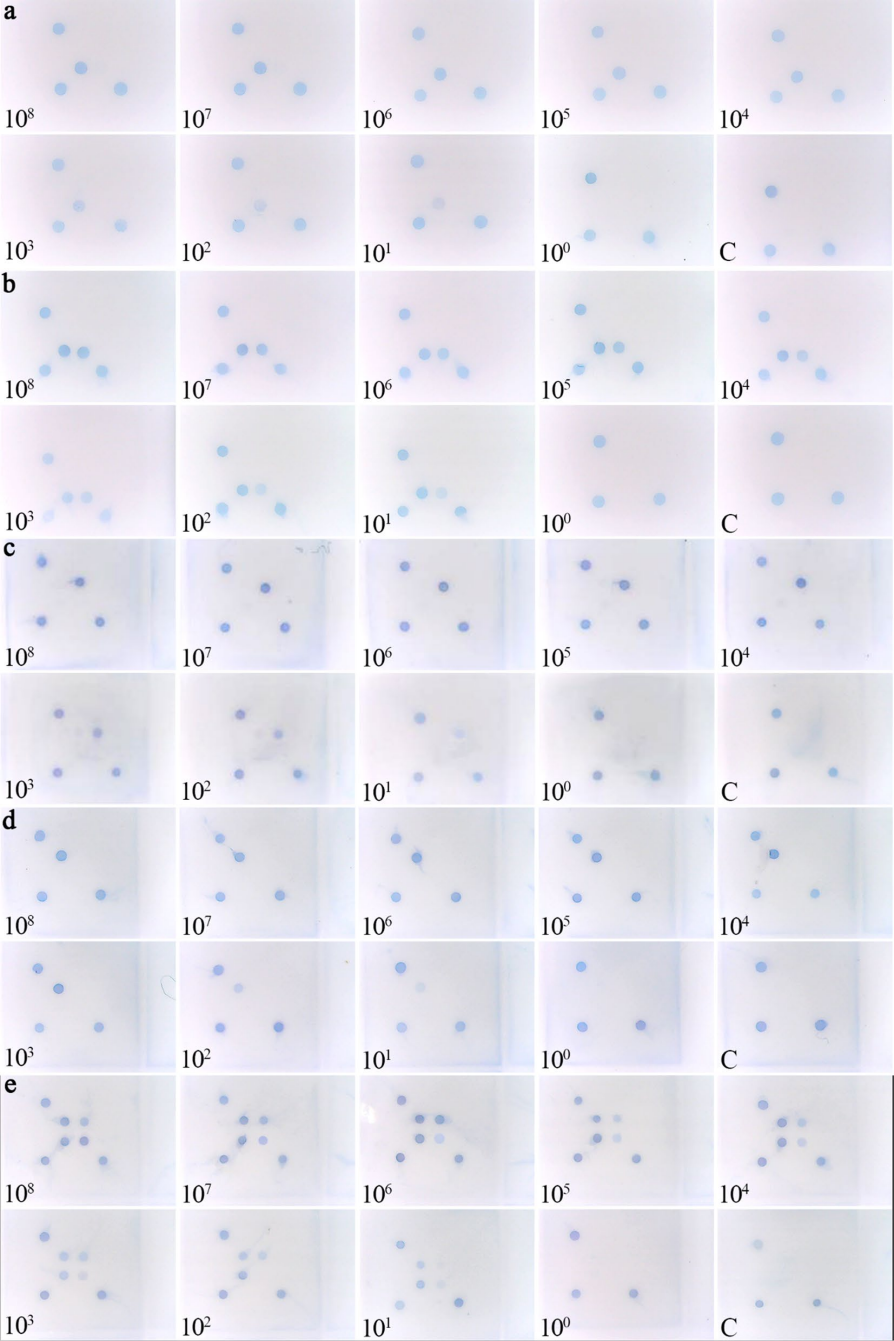
The sensitivity test results of the gene microarray assay

Four positive plasmids, CSFV-W-p, CSFV-(W + V)-p, ASFV-p, and APPV-p, were tested with series dilutions ranging from 10^8^ copies/µL to 10^–1^ copies/µL. The LOD was 6.98 copies/µL for CSFV-W–p (Fig. 3a), 6.92 copies/µL for CSFV-(W + V)-p (Fig. 3b); 2.56 × 10 copies/µL for ASFV-p (Fig. 3c); and 1.8 × 10 copies/µL for APPV-p (Fig. 3d), respectively. The mixture of the four plasmids was also tested for the quadruple assay with 1 µL of each plasmid, and the concentration ranged from 10^8^ copies/µL to 10^–1^ copies/µL. The LOD of the quadruplet test was illustrated as the minimum amount of plasmids used to ensure all four target genes were detected. The results showed that the minimum detection limit of the quadruple assay was 2.9 × 10 copies/µL of each plasmid (Fig. 3).

Four positive plasmids CSFV-W-p(a), CSFV-(W + V)-p(b), ASFV-p(c), and APPV-p(d) were tested separately and jointly (e) using plasmids specific for each antigen with serious dilutions ranging from 10^8^ copies/µL to 10^–1^ copies/µL. C was the negative control.

### Tests using clinical samples

In recent years, 219 clinical samples collected were collected by our laboratory. We tested these samples by this assay and by standard methods for CSFV, ASFV, and APPV for simultaneous comparison. The results showed that 54 samples were positive for CSFV-W, 41 were positive for CSFV-V, nine were positive for ASFV, 10 were positive for APPV, and 106 were negative. CSFV RT-nPCR followed by sequencing showed 54 samples were wild-type CSFV strains, 41 were CSFV vaccine strains. Nine samples were positive for the ASFV qPCR method and 10 were positive for APPV conventional RT-PCR method. The remaining 106 samples were negative for the three methods. The results of the gene chip were 100% consistent with the other three standard detection methods, indicating that the established gene chip assay can rapidly and accurately identify CSFV-W, CSFV-V, ASFV and APPV in the field, and is practical for differential diagnosis, surveillance and elimination of CSFV, ASFV and APPV (Table 5).

**Table 5:** Results for clinical samples

Sample type	Positive samples/rate by gene chip	Positive samples/rate by standard methods
CSFV-W	54/24.7%	54/24.7%
CSFV-V	40/18.3%	40/18.7%
ASFV	9/4.1%	9/4.1%
APPV	10/4.6%	10/4.6%
Negative samples	106	106
In total	219	219
Coincidence rate %	100%	

## Discussion

Before 2016, CSF in China was under control with the effort of the compulsory vaccination policy of the C strain vaccine [20, 21]. However, with the development of intensive farming, many large-scale pig farms have been built in China. In addition, the compulsory vaccination policy has been replaced by widespread vaccination since July 2016, which greatly impact on the CSF situation [22]. Chronic and atypical CSF has become dominant in epidemic outbreaks, characterized as sporadic, vulnerable at a young age, persistent infection, complex onset, and immune tolerance, which have brought new challenges to the prevention and control of CSF [23, 24]. In particular, it is clinically difficult to distinguish between infection and vaccination, which brings new challenges to the prevention, control, and elimination of CSFV in China. The outbreaks of ASF in 2018 in China have devastatingly impacted the country's pig industry [5]. With the rapid response of our government and a series of precise control policies, the outbreak has been effectively controlled and the pig industry has been recovering in an orderly way [25]. In 2021, however, new situations for the ASF epidemic in China emerged, which showed reduced mortality, and atypical clinical signs, and some "natural variant strains" showed no hemadsorption (HAD) [26]. These natural variant ASFV strains caused subclinical symptoms, which were difficult to identify and detect at the early stage and can be easily confused with other diseases, leading to problems in differential diagnosis and prevention and control of ASFV [27]. APPV, commonly known as "piglet shivering disease" or "jumping disease" is a disease in which piglets’ exhibit paroxysmal muscle movements in the head, limbs, and other parts of the body [28]. It can cause piglets difficulties standing, blocked suckling and even death. It is estimated that the number of piglets weaned by APPV-infected sows could reduce by 10%, and the mortality rate of newborn piglets affected by APPV could rise to 30% due to malnutrition [29]. APPV is widespread in pig herds throughout China and the world, posing a severe threat to the pig industry [30]. CSFV, ASFV, and APPV have become three important contagious, virulent infectious diseases in China, which show similar clinical signs and are difficult to distinguish from each other. It is, therefore, critical to developing a simple, rapid, specific, and sensitive assay to differential diagnose these three diseases.

In the present study, we reported a gene microarray assay for the detection of ASFV, CSFV, and APPV, which is the first assay to simultaneously detect the three swine diseases. The specificity of the products, the concentration of each reagent, and, most importantly, the possible interference among multiple primers and probes can significantly impact on the development of multiplex PCR. A duplex real-time PCR assay for CSF and ASF detection showed a limit of close to 100 copies per reaction for CSFV [31]. In the present study, the sensitivity of this assay is 6.98 copies/µL and 69.2 copies/µL for CSFV-W and CSFV-V, respectively, which is more sensitive than real-time multiplex PCR [31], demonstrating the advantage of the gene chip assay in sensitivity. In addition, this assay can detect four pathogens of interest in one sample simultaneously all at once and 48 samples simultaneously, which is much time-saving than traditional virus isolation. Furthermore, the results of this assay could be visualized. In the current study, after looking through the 5′UTR gene sequences of 30 CSFV field strains preserved in our laboratory and 10 published strains on NCBI and 4 vaccine strains, we found that the 5′UTR was highly conserved among them. Many CSFV RT-PCR diagnostic methods also chose this region for primer design [32, 33]. NS5B is an RNA polymerase involved in viral genome replication and is one of the popular targets for CSFV genotyping. We found a base difference between the vaccine and wild strains in the NS5B region which was then used for MGB probe designed to specifically bind to the NS5B gene of CSFV vaccine strains. It is not only practical to the C strain, which is widely used in China, but also applicable to the CSFV low-temperature mutagenesis vaccine (Thiveosal strain).

The biological reaction between samples and the gene chip is critical for the successful detection and subsequent analysis of the gene chip assay. The size of the gene probe and the length of PCR products on the microarray are also important factors affecting the hybridization signal of the microarray [34]. Therefore, the length of the probes designed in this experiment is less than 30 bp, and the length of the PCR products is less than 100 bp, thus ensuring a stable and precise signal response. To facilitate the interpretation of the microarray results, primers were labeled with biotin that has a good affinity with streptavidin. After quadruple PCR amplification, the products were combined with the probe on the microarray and reacted with the HRP-labelled streptavidin. Conventional gene microarrays usually use aldehyde-based slides as support and take longer for detection. This assay uses "0 + X" nano-membranes (0 for zero background and X for various probes) supported by high-topping materials, which significantly reduces the reaction time compared to conventional one, resulting in significant time and cost savings. The hybridization process is simple. The results are entirely consistent with those of national standard assays. The assay also allows adding other swine infectious diseases other than the three we target in this study. Therefore, the gene chip assay is potentially used in diagnosing and surveillance for swine and other animal diseases.

## Conclusion

In this study, a novel gene chip assay was developed for rapid clinical identification of CSFV wild type strains and vaccine strains, ASFV and APPV with high specificity and sensitivity. This assay provides a practical, simple, economical and reliable way for the rapid and accurate diagnosis of CSFV, ASFV and APPV, and also provides a platform and new thoughts for multiple animal diseases detection using gene chips.

## Data Availability

All data generated or analyzed during this study are included in this published article.

## Abbreviations

ARMS-PCR: Amplification refractory mutation system PCR principle
ASFV: African swine fever virus
APPV: Atypical porcine pestivirus
BVDV: Bovine viral diarrhea virus
CSFV-W: Classical swine fever virus wild type
CSFV-V: Classical swine fever virus vaccine strains
HAD: Hemadsorption
IVDC: Institute of China veterinary drug control
JEV: Japanese encephalitis virus
MGB: Minor groove binder
NCBI: National center for biotechnology information
NS5B: Nonstructural protein 5B
PCV1: Porcine circovirus 1
PCV2: Porcine circovirus 2
PEDV: Porcine epidemic diarrhea virus
PRRSV: Porcine reproductive and respiratory syndrome virus
PRV: Pseudorabies virus
RT-PCR: Reverse-transcription polymerase chain reaction
TGEV: Transmissible gastroenteritis virus
PPV: Porcine parvovirus
5′UTR: Five prime untranslated region
